# Mechanism of Action of Oral *Salmonella*-Based Vaccine to Prevent and Reverse Type 1 Diabetes in NOD Mice

**DOI:** 10.3390/vaccines12030276

**Published:** 2024-03-06

**Authors:** Jacob Cobb, Jeffrey Rawson, Nelson Gonzalez, Mahmoud Singer, Fouad Kandeel, Mohamed I. Husseiny

**Affiliations:** 1 Department of Translational Research & Cellular Therapeutics, Arthur Riggs Diabetes & Metabolism Research Institute, Duarte, CA 91010, USAfkandeel@coh.org (F.K.); 2 Beckman Research Institute, City of Hope National Medical Center, Duarte, CA 91010, USA; 3 School of Medicine, University of California Irvine, Irvine, CA 92697, USA; singermk@hs.uci.edu

**Keywords:** *Salmonella*-based vaccine, regulatory cells, tolerogenic dendritic cell (Tol-DC), type 1 diabetes (T1D), tolerance, oral vaccination

## Abstract

A combination therapy of preproinsulin (PPI) and immunomodulators (TGFβ+IL10) orally delivered via genetically modified *Salmonella* and anti-CD3 promoted glucose balance in in NOD mice with recent onset diabetes. The *Salmonella* bacteria were modified to express the diabetes-associated antigen PPI controlled by a bacterial promoter in conjunction with over-expressed immunomodulating molecules. The possible mechanisms of action of this vaccine to limit autoimmune diabetes remained undefined. In mice, the vaccine prevented and reversed ongoing diabetes. The vaccine-mediated beneficial effects were associated with increased numbers of antigen-specific CD4^+^CD25^+^Foxp3^+^ Tregs, CD4^+^CD49b^+^LAG3^+^ Tr1-cells, and tolerogenic dendritic-cells (tol-DCs) in the spleens and lymphatic organs of treated mice. Despite this, the immune response to *Salmonella* infection was not altered. Furthermore, the vaccine effects were associated with a reduction in islet-infiltrating lymphocytes and an increase in the islet beta-cell mass. This was associated with increased serum levels of the tolerogenic cytokines (IL10, IL2, and IL13) and chemokine ligand 2 (CCL2) and decreased levels of inflammatory cytokines (IFNγ, GM-CSF, IL6, IL12, and TNFα) and chemokines (CXCL1, CXCL2, and CXCL5). Overall, the data suggest that the *Salmonella*-based vaccine modulates the immune response, reduces inflammation, and promotes tolerance specifically to an antigen involved in autoimmune diabetes.

## 1. Introduction

Type 1 diabetes (T1D) is a chronic autoimmune disorder that leads to the destruction of pancreatic insulin-producing beta-cells [1,2,3]. However, there are no accepted therapies that restore tolerance to inciting autoantigens to resolve the disease. Insulin is one of the dominant antigens in diabetic animals [4] and individuals with T1D [5,6,7]. To reverse T1D, one strategy sought to induce tolerance to diabetogenic immune cells without using immunosuppression [8]. Taking a different approach, we used an oral live attenuated *Salmonella*-based antigen-specific vaccine against diabetes [9,10,11,12,13,14]. However, the mechanisms behind the vaccine effects remain to be determined.

In preclinical studies, non-pathogenic *Salmonella* was efficient and safe in delivering antigens orally [9,10,11,12,13,14,15,16,17,18,19,20]. A benefit of the recombinant *Salmonella*-based vaccine is that it delivers antigen-expressing plasmid to phagosomes of the gut-associated lymphoid tissue (GALT). This is where antigen presenting cells (APCs) reside and produce *Salmonella*-containing vacuoles (SCV) [19,21]. Related to this, we found that the APC processing of antigens and subsequent presentation to T-cells was associated with CD8 and CD4 T cell-mediated immune responses in animals with cancers [16,22,23].

In experimental models of diabetes, a tolerating immune response against autoantigens prevented further beta-cell destruction [8,24]. Approaches to generate tolerance in pre-clinical T1D included the deletion of alloantigen [25], tetramer and peptide therapy [26], depletion of effector cells [27], inhibition of immune activation molecules, expression of cell membrane suppressor molecules (PD-1, CTLA-4, and CD47) [28], and expansion of regulatory T-cells [29].

Regulatory CD4^+^ CD25^+^ Foxp3^+^ T-cells (Tregs) express high levels of CD25, which helps to maintain tolerance against self- and non-self-antigens in T1D [30]. Tregs limit the proliferation and function of effector T-cells (Teffs) and APCs [31,32]. Also, Tregs express cell surface inhibitory molecules such as CTLA-4, LAG3, PD-1, and CD47, secrete the anti-inflammatory cytokines IL10, IL35, and TGFβ [33], and kill effector cells with perforin and granzyme [34]. Nonetheless, in clinical T1D, Tregs were defective in number [35,36] and function [37].

Dendritic cells (DCs) activate autoreactive T-cells in NOD mice to present islet self-autoantigens while altering Treg function. The later interaction modifies self-antigen presentation and drives Tregs to limit IFNγ-producing NK cells, thus permitting tissue injury from autoreactive CD4^+^ Teffs [24]. DCs are needed for the induction of effector immune responses and tolerance [38]. DCs interact with Tregs in the GALT to deter the normal differentiation of Tregs and contribute to immune dysfunction in T1D. Such DCs are known as tolerogenic DCs (tol-DCs). This interaction results in the subsequent emergence of CTLA-4 or PD-L1 which are normally expressed in Foxp3^+^ Tregs, Th3 regulatory cells, Treg 17 cells, and type 1 regulatory T-cells (Tr1), all of which may diminish the capacity of effector T-cells [9,39,40,41,42,43,44,45].

Peripheral immune tolerance can emerge with chronic exposure to antigens and the activation of Tr1 cells in the presence of IL10 [11]. Tr1 cells lack Foxp3 and produce regulatory cytokines like IL10 and express inhibitory cell surface receptors to suppress T-cells and modulate APCs [46,47].

The *Salmonella* vaccine was designed to induce the expression of the diabetogenic autoantigen preproinsulin (PPI) and immune regulating TGFβ and IL10 [9,10,11]. When given with anti-CD3 mAb to NOD mice with auto-immune diabetes, the vaccine promoted beta cell survival, reduced inflammation, and prevented and reversed disease [11,12,13]. The possible mechanisms of action of the *Salmonella*-based vaccine to limit autoimmune diabetes are not fully known. Herein, mechanistic characterization of the *Salmonella*-based vaccine in the prevention and reversal of diabetes in NOD mice was undertaken.

## 2. Materials and Methods

### 2.1. Preparation of the Salmonella Vaccine

Attenuated *Salmonella* were transfected with appropriate plasmids to force the expression of antigens and immune regulators [9,10,11,12,13]. The bacteria were cultured in growth media and resuspended in 5% sodium bicarbonate for oral administration as published [10,11,12,13].

### 2.2. Animal Vaccination

Young female NOD/ShiLtJ (NOD) and NOD.*Cg-Prkdc^scid^ Il2rg^tm1Wjl^*/SzJ (NSG) mice (Jackson Laboratory, Bar Harbor, ME, USA) were housed at City of Hope and orally vaccinated on days 0 and 7 and treated for 5 days with hamster anti-CD3 mAb (2.5 μg i.p./mouse) [10,11,12]. Control mice were administered 5% sodium bicarbonate solution.

### 2.3. Flow Cytometry

Spleens, mesenteric lymph nodes (MLNs), pancreatic lymph nodes (PLNs), and Peyer’s patches (PPs) taken from mice a month after treatment were processed into single cells. LIVE/DEAD Fixable Blue Dead Cell Stain kit (Invitrogen, Waltham, MA, USA) was used to determine cell viability. Tregs were stained with FITC anti-mouse CD4 (RM4-5), Brilliant Violet 650 anti-mouse CD8a (53-6.7), PE anti-mouse FOXP3 (MF-14), APC anti-mouse CD25 (PC61), and matching isotype controls FITC Rat IgG2a,κ, Brilliant Violet 650 Rat IgG2a,κ, PE Rat IgG2b,κ, and APC rat IgG1,λ.

Tr1 cells were identified using APC anti-mouse LAG-3 (C9B7W), PerCP Cy5.5 anti-mouse CD49b (HMα2), and matching isotype APC Rat IgG1,κ, PerCP/Cy5.5 Armenian Hamster IgG.

DCs were stained with FITC anti-mouse MHC class II (10-3.6), APC anti-mouse CD11c (N418), PE/Cy7 anti-mouse CD11b (M1/70), Brilliant Violet 650 anti-mouse CD45R/B220 (RA3-6B2), Brilliant Violet 510 anti-mouse Gr-1 (RB6-8C5), and PE anti-mouse CD80 (16-10A1), Brilliant Violet 711 anti-mouse CD86 (GL1) (Becton Dickinson Biosciences, Franklin Lakes, NJ, USA), and matching isotype controls FITC Mouse IgG2a,κ, APC Armenian Hamster IgG, PE/Cy7 Armenian Hamster IgG, Brilliant Violet 650 anti-mouse CD8a (53-6.7), Brilliant Violet 510 Rat IgG2b,κ, PE Armenian Hamster IgG, and Brilliant Violet Rat IgG2a,κ (BD Biosciences, Franklin Lakes, NJ, USA).

Cells were stained for activation markers using PE anti-mouse/human CD44 (IM7), PE/Cy7 anti-mouse CD69 (H1.2F3), and matching isotype controls PE Rat IgG2b,κ, PE/Cy7 Rat IgG2b,κ. Antibodies were obtained from Biolegend (San Diego, CA, USA). Cells were analyzed with a BD Fortessa flow cytometer (BD Biosciences) and FlowJo software 10.4.

### 2.4. In Vitro Antigen-Specific Suppression Assay

To incite an immune response to an insulin-relevant antigen, we gave the insulin peptide B_9-23_ emulsified in an equal amount with complete Freund adjuvant to young nondiabetic NOD mice. At ten days following antigen challenge, mice were humanely euthanized and CD4^+^ splenocytes were isolated and further purified using a CD4^+^ CD25^+^ regulatory T-cell isolation kit (Miltenyi Biotec, 130-091-041, San Diego, CA, USA) to provide a homogenous pool of CD4^+^ CD25^−^ responder T-cells (Tresp). These cells were then tagged with a vital stain Cell Trace Violet (CTV) [48]. Tresp cells were mixed with splenic APCs from NOD/SCID γc^−/−^ mice (1: 2 ratio) pulsed with 10 µg/mL of insulin B_9-23_ peptide at several ratios (0:1, 1:1, 1:2, and 1:8) of CD4^+^ CD25^+^ T-cells isolated from spleens of vaccine- and control-treated mice. CD4^+^ CD25^+^ T-cells (Treg) were isolated from pooled splenocytes at day 30 post-treatment using negative selection for CD4^+^ followed by positive selection for CD25^+^ using a CD4^+^ CD25^+^ regulatory T Cell Isolation Kit (Miltenyi Biotec). CD4 Tresp cells positive for CTV and with surface expression of CD44 and CD69 (activation markers) were characterized for proliferation using FlowJo software 10.4 [49,50,51]. Supernatants were analyzed for levels of IL10, IL2, and IFNγ using a Mouse Quantikine ELISA Kit (R&D Systems, Minneapolis, MN, USA) [16,48].

### 2.5. Adoptive Transfer of Diabetes

Splenocytes (1 × 10^6^ cells) were isolated from NOD mice with T1D and given to immune-incompetent NSG mice [11]. In other studies, 1 × 10^6^ splenocytes from control- or vaccine-treated mice were depleted of CD4 cells and given to NSG mice.

A month after treatment, splenic or MLNs DCs were isolated from mice using PE-conjugated CD11c antibody (Miltenyi, 130-110-701) followed by anti-PE microbeads (Miltenyi Biotec, 130-091-041). The DCs (1 × 10^6^) were then given to non-diabetic NOD mice and their blood glucose levels were tracked.

### 2.6. ELISA and Cytokine Measurement

Soluble circulating cytokines were quantified by a MILLIPLEX MAP Mouse High Sensitivity T Cell Premixed Panel—Immunology Multiplex Assay (MilliporeSigma, Burlington, MA, USA) and a Bio-Plex analyzer (Bio-Rad, Hercules, CA, USA).

From treated mice, splenocytes (5 × 10^5^ cells) were incubated with insulin peptide B_9-23_ for 72 h. Cell-free supernatants were tested for IL10, IL2, and IFNγ with a Mouse Quantikine ELISA Kit (R&D Systems, Minneapolis, MN, USA).

### 2.7. Gene Expression

Splenocytes, CD11c^+^ cells (DCs), CD4^+^ CD25^+^ cells (Tregs), and CD4^+^ CD49b^+^ (Tr1) cells were processed and RNA was prepared using the DirectZol kit (Zymo Research, Orange, CA, USA). A Nanodrop 2000 (Thermofisher Scientific, Irwindale, CA, USA) was used to determine RNA concentration. cDNA was prepared via a qScript cDNA SuperMix kit (Quantabio, Beverly, MA, USA). qPCR analysis was performed using TaqMan gene expression assays (Applied Biosystems, Foster City, CA, USA). The following commercial primers and probes were used: CD274 (Mm03048248_m1), CTLA-4 (Mm00486849_m1), IDO (Mm00492590_m1), AhR (Mm00478932_m1), IL27 (Mm00461162_m1), and CD209A (Applied Biosystems). Relative changes in mRNA to the control gene (TATA box binding protein, Tbp (Mm01277042_m1) were calculated using the following formula ΔCt = Ct*_Target_* − Ct*_Reference_*. The expression level was 2^−ΔCt^.

### 2.8. Statistical Analyses

Differences in cell numbers, cytokine levels, and mRNA levels were assessed for significance using unpaired and Mann–Whitney *t* tests. To compare combination treatments to the control, the Dunnett’s test was used. Kaplan–Meier graphs and the Mantel–Cox log-rank test were used to look at the incidence of diabetes among treated mice. Data from FACS analysis were compared using a two-way ANOVA test. Significance was assumed if the *p* < 0.05. GraphPad Prism 10 software was used for the calculations.

## 3. Results

### 3.1. A Salmonella-Based Vaccine Reverses Diabetes in Diabetic Mice

**Figure 1:**
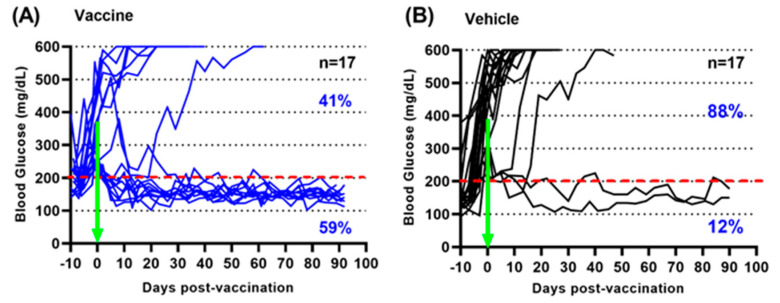
(**A**) *Salmonella*-based vaccine reverses new-onset diabetes in mice. Recently hyperglycemic NOD mice were given the *Salmonella* vaccine (*n* = 17, (**A**)) or the vehicle (*n* = 17, (**B**)) and blood glucose levels were determined for 3 months. Blood glucose levels above the hashed red line are taken as abnormal. Initiation of treatment is highlighted by the green arrow.

An oral *Salmonella*-based vaccine was developed to treat pre-clinical diabetes [11,13]. Newly hyperglycemic NOD mice were vaccinated orally with the *Salmonella*-based vaccine. Fifty-nine percent of diabetic mice treated with the oral vaccine became euglycemic and stable by 3 months post-treatment (Figure 1A), while 88% of vehicle-treated diabetic mice were hyperglycemic at 3 months (Figure 1B).

### 3.2. A Salmonella-Based Vaccine Increases Tolerogenic Cytokines and Decreases Pro-Inflammatory Cytokines without Altering the Immune Response to Salmonella

**Figure 2:**
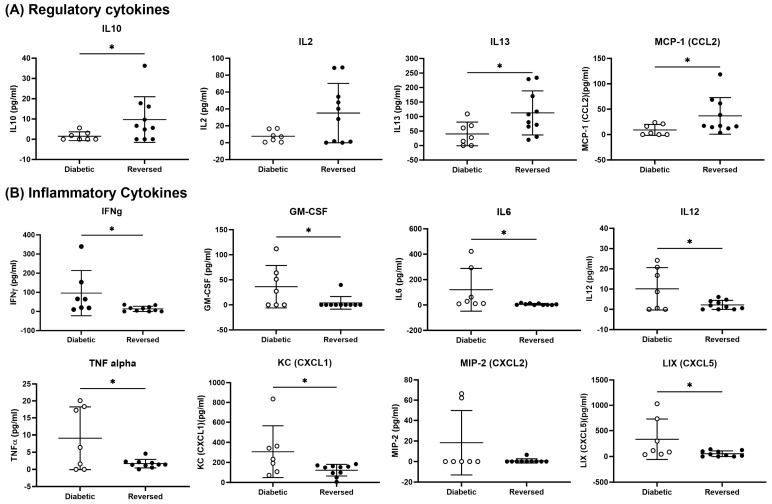
A *Salmonella*-based vaccine increases serum tolerogenic cytokines and decreases pro-inflammatory cytokines in treated diabetic NOD rendered normoglycemic. Serum was collected from vaccine-treated mice and cytokines were quantified using a multiplex assay. (**A**) Levels of regulatory cytokines IL10, IL2, IL13, and CCL2. (**B**) Levels of pro-inflammatory cytokines IFNγ, GM-CSF, IL6, IL12, TNFα, CXCL1, CXCL2, and CXCL5. Data presented as means ± SD from vaccine-treated mice: 7 diabetic (non-responders) and 10 reversed (responders). Significant differences between diabetic and reversed mice were determined by the unpaired *t* test (* *p* < 0.05).

Cytokines play crucial roles in regulating the interaction between beta-cells and immune cells in the development of T1D. For example, cytokines induced regulatory functions to restore immune tolerance and prevent the destruction of beta-cells [33]. In addition, other cytokines induced inflammatory functions that promoted the differentiation and function of immune cells, leading to T1D onset and progression [33]. To assess if the vaccine induced tolerance, circulating tolerogenic and inflammatory cytokines were quantified in serum from vaccinated mice (*n* = 17) and serum levels from diabetic (non-responders) and reversed (responders) mice were compared (Figure 2). Levels of tolerogenic cytokines IL10, IL2, IL13, and MCP-1 (CCL2) were increased in the serum of reversed (responders) compared with diabetic mice (non-responders) (unpaired *t* test, *p* = 0.045, 0.06, 0.04, and *p* = 0.04) (Figure 2A). Conversely, pro-inflammatory cytokines IFNγ, GM-CSF, IL6, IL12, and TNFα were lower in the serum of reversed (responders) compared with diabetic animals (non-responders) (unpaired *t* test, *p* = 0.043, *p* = 0.04, *p* = 0.04, 0.03, and *p* = 0.02) (Figure 2B). Furthermore, lower amounts of inflammatory chemokines CXCL1, CXCL2, and CXCl5 were detected in the serum of vaccine-treated mice (*p* = 0.04, *p* = 0.09, and *p* = 0.04) (Figure 2B). In addition, circulating tolerogenic and inflammatory cytokines were quantified in serum from vaccinated mice (*n* = 17) and compared to levels in serum from vehicle-treated mice (*n* = 17) (Figure S1). Regulatory cytokines IL10, Il2, IL13, and CCL2 were increased in vaccine- compared to vehicle-treated mice (Figure S1A). No differences were seen in inflammatory cytokines among the treatment groups (Figure S1B). The increase in IL10 levels is likely not the result of IL10 being over-expressed by the *Salmonella* vector since IL10 levels were measured 3 months after *Salmonella* was cleared from the animals. LPS antibodies against *Salmonella* were increased in the serum of *Salmonella*-infected animals as well as in the serum of vaccine- compared to vehicle-treated animals (one–way ANOVA, *p* < 0.0001, and *p* < 0.0001) (Figure S2).

### 3.3. A Salmonella-Based Vaccine Induces Regulatory T-Cells

**Figure 3:**
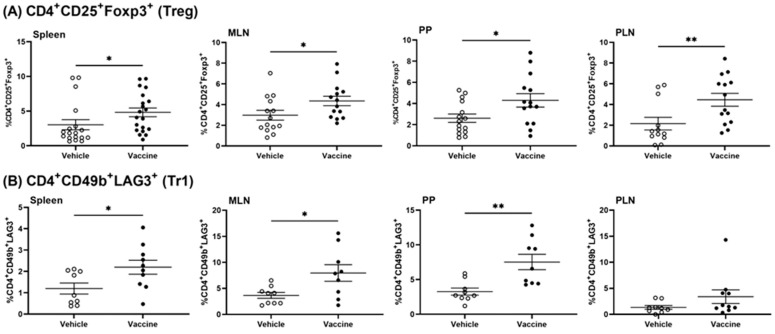
A *Salmonella*-based vaccine increases regulatory T-cells in lymphatic tissues. NOD mice received the vaccine or vehicle, and a month later immune cells from spleens, MLNs, PPs, and PLNs were analyzed via FACS for CD4^+^ CD25^+^ Foxp3^+^ (Treg, (**A**)) cells and CD4^+^ CD49b^+^ LAG3^+^ (Tr1, (**B**)) cells. Data as means ± SD from 3 independent experiments. Significant difference between vaccine- and vehicle-treated groups was determined by unpaired *t* test (* *p* < 0.05, ** *p* < 0.01).

To test whether the *Salmonella*-based vaccine induced tolerance via Tregs, we examined the type and number of Tregs present in vaccine- and vehicle-treated animals using flow cytometry. To identify the specific cell types that were involved in the vaccine-mediated effects, the frequency of regulatory T-cells in lymphatic organs (spleens, MLNs, PPs, and PLNs) of vaccine-treated mice was characterized (Figure S3). Greater percentages of regulatory CD4^+^ CD25^+^ Foxp3^+^ (Treg) cells were present in the spleens, MLNs, PPs, and PLNs of vaccinated mice compared to vehicle-treated mice (Mann–Whitney test, *p* = 0.019, *p* = 0.032, *p* = 0.049, and *p* = 0.004) (Figure 3A).

Additionally, CD4^+^ CD49b^+^ LAG3^+^ type 1 regulatory (Tr1) cells were increased in spleens, MLNs, PPs, and PLNs of vaccine- compared to vehicle-treated mice (Mann–Whitney test, *p* = 0.037, *p* = 0.03, *p* = 0.003, and *p* = 0.11) (Figure 3B).

### 3.4. A Salmonella-Based Vaccine Induces Functional Tregs

We next evaluated whether Tregs in vaccine-treated mice were functional. The in vitro function of the CD4^+^ CD25^+^ Tregs from treated mice was assessed by mixing them with CD4^+^ CD25^−^ Tresp cells from NOD mice. Confirming immune function, the Tregs from vaccine-treated mice limited the proliferation and activation of Tresp cells, as shown by less CD69 and CD44 expression (Figure S4A–C). In addition, the conditioned medium from vaccine-treated Tregs had more IL10 (Figure S4D) and less IFNγ (Figure S4F) although IL2 was not different (Figure S4E).

### 3.5. A Salmonella-Based Vaccine Induces Antigen-Specific Suppressor Tregs

**Figure 4:**
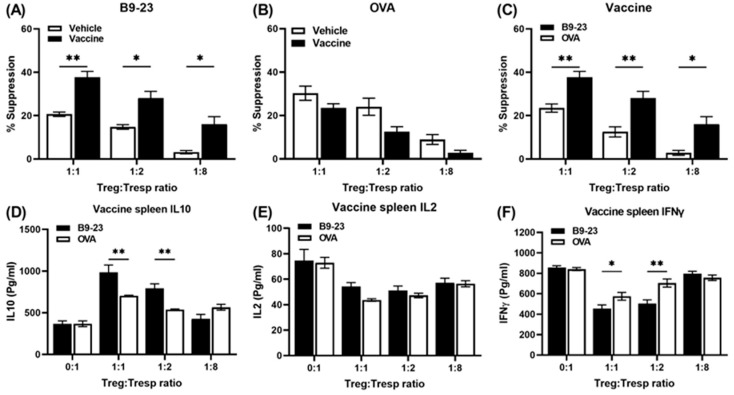
A *Salmonella*-based vaccine induces antigen-specific suppressor Tregs. CD4C^+^ D25^−^ Tresps isolated from splenocytes of B_9-23_ challenged mice were dye-labeled, cultured with splenic APCs pulsed with insulin peptide B_9-23_ (10 mg/mL), and co-cultured for 4 days with different ratios of CD4^+^ CD25^+^ Tregs. Percent suppression activity of Tregs from vaccine- and vehicle-treated mice after stimulation with B_9-23_ (**A**), OVA peptide (**B**), or vaccine-treated mice after stimulation with insulin B_9-23_ and OVA peptide (**C**). Measurement of IL10 (**D**), IL2 (**E**), and IFNγ (**F**) in conditioned medium by ELISA assay after stimulation of cells with peptides B_9-23_ and OVA. The data shown are the average ± SD from two separate experiments. Statistical analysis was performed by two-way ANOVA (* *p* < 0.05, ** *p* < 0.01).

To assess whether Tregs from vaccine-treated mice responded specifically to the autoantigen, we performed an in vitro antigen-specific suppressor assay. For this, insulin B_9-23_ specific Tresp cells were generated by immunizing mice with the insulin peptide B_9-23_. As expected, the Tresp cells grew more when re-exposed to the insulin peptide (Figure S4A). In culture, the Tregs from vaccine-treated mice suppressed the proliferation of Tresp cells exposed to the insulin peptide (Figure 4A). Conversely, Tregs from both vaccine- and vehicle-treated mice were equally suppressive toward OVA-stimulated Tresps (Figure 4B). CD4^+^ CD25^+^ Tregs from vaccine-treated mice suppressed insulin peptide-stimulated Tresp cells more potently than Tresp cells in the presence of OVA peptide (Figure 4C). IL10 (Figure 4D) but not IL2 (Figure 4E) was increased in response to B_9-23_, more in Tregs from vaccine-treated mice than in response to OVA peptide. Levels of IFNγ were lower in cultured Tregs from vaccine-treated mice incubated with the peptide B_9-23_ (Figure 4F).

### 3.6. A Salmonella-Based Vaccine Induces Tolerogenic Dendritic Cells (tol-DCs)

**Figure 5:**
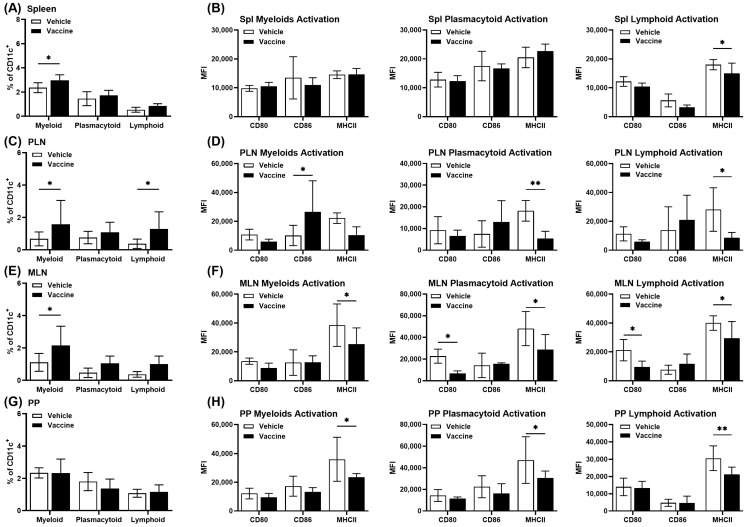
Markers of activation among DCs from treated mice. NOD mice were given the vaccine or vehicle and a month later DCs from lymphatic organs were characterized as myeloid (CD11c^+^ CD11b^+^), plasmacytoid (CD11c^+^ B220^+^), and lymphoid (CD11c^+^ CD8^+^) DCs. The expression levels of CD80, CD86, and MHC class II were demarcated in DCs. Relative numbers of DCs were found in spleens (**A**,**B**), PLNs (**C**,**D**), MLNs (**E**,**F**), and PPs (**G**,**H**). The data shown are the average percent mean fluorescence intensity (MFI) ± SD from two independent experiments. (* *p* < 0.05, ** *p* < 0.01).

DCs are needed for the induction of effector immune responses and tolerance [38]. DCs are also a maintenance factor for Tregs [52]. Our previous study showed that CD11c^+^ DCs were significantly increased in lymphatic organs from vaccine- compared to vehicle-treated animals [12]. DCs subsets in different lymphoid organs were characterized using flow cytometry (Figure S5). A higher percentage of myeloid DCs (mDCs) (CD11c^+^ CD11b^+^) were detected in spleens of vaccine- compared to vehicle-treated mice whereas no changes in the plasmacytoid DCs (pDCs) (CD11c^+^ B220^+^ GR1^+^) and lymphoid DCs (CD11c^+^ CD8^+^) were found (Figure 5A). Lymphoid DCs in spleens from vaccine-treated mice showed lower expression of MHCII and co-stimulatory molecules CD80 and CD86, but these changes were not found in mDCs and pDCs (Figure 5B). In PLNs, a higher percentage mDCs and lymphoid DCs were detected in vaccine-treated mice (Figure 5C). The pDCs and lymphoid DCs in PLNs showed less expression of MHCII and co-stimulatory CD80 and CD86, but no changes were noted in mDCs (Figure 5D). A higher percentage of mDCs and no changes in pDCs and lymphoid DCs were detected in MLNs of vaccine-treated mice (Figure 5E), with less expression of MHCII and CD80 and CD86 in all subsets of DCs (Figure 5F). No changes in the percentage of all subsets of DCs were detected in PPs of vaccine-treated mice (Figure 5G), with less expression of MHCII, CD80, and CD86 in all subsets of DCs (Figure 5H).

### 3.7. Tolerogenic DCs Contribute to Diabetes Prevention by a Salmonella-Based Vaccine

**Figure 6:**
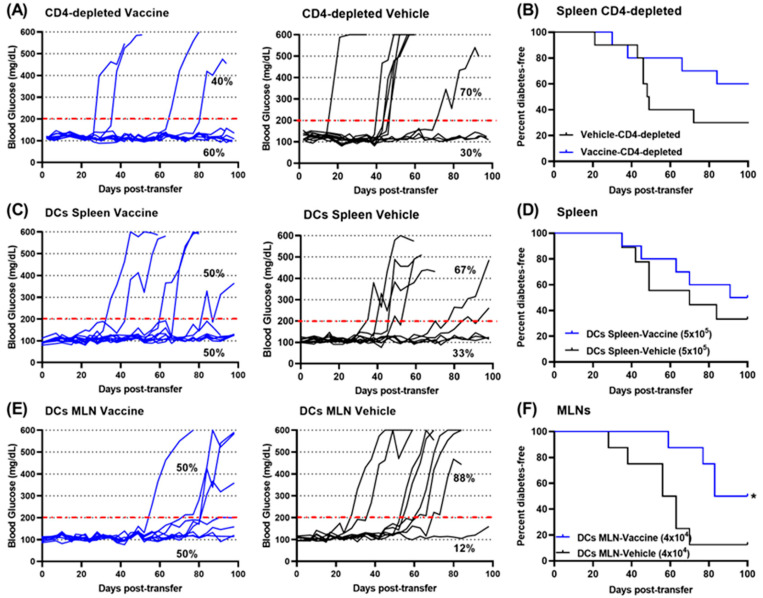
DCs from vaccinated mice are anti-diabetogenic. A month after treatment, pooled splenocytes were obtained from NOD mice and given to NSG mice (**A**,**B**) or to non-diabetic NOD mice (**C**,**D**). DCs derived from the MLNs of treated mice were given to NOD mice (**E**,**F**). Blood glucose levels were tracked (**A**,**C**,**E**). Log-rank plot of the percentage of recipient mice that remained diabetes-free over time (**B**,**D**,**F**). Log-rank (Mantel–Cox) test of differences between the vaccine- and vehicle-treated mice was significant. The dotted red line indicates the threshold blood glucose level of 200 mg/dL. (* *p* < 0.05).

Previously, we observed that the transfer of splenocytes from diabetic NOD mice into immune-deficient NSG mice resulted in the NSG mice developing diabetes. These published results confirmed that the transfer of cells among mice permits the in vivo testing of immune cell function [11]. First, we inquired if cells other than CD4^+^ cells played a part in the effect of the vaccine. We found that splenocytes from vaccinated mice that were rendered deficient in CD4 cells promoted diabetes much less than similar cell from vehicle-treated mice (Figure 6A,B).

Next, it was reasonable to consider that DCs could be involved in the vaccine effect. This was tested by adoptive transfer. DCs were isolated from the spleens and MLNs from vehicle- and vaccine-treated mice on day 30 post-treatment and transferred into 8-week-old normoglycemic NOD mice. The transfer of DCs from spleens of vaccine-treated animals protected 50% of the mice from diabetes for 100 days (Figure 6C), whereas only 33% of mice given DCs from vehicle-treated mice were protected (Figure 6C). In other words, the splenic DCs from vaccine-treated mice protected mice from developing diabetes better than the splenic DCs from vehicle-treated mice (Figure 6D). MLN DCs from vaccine-treated mice protected half of recipient mice from diabetes, whereas only 12% of mice given MLN DCs from vehicle-treated mice were protected (Figure 6F). This suggests that DCs from MLNs from vaccine-treated mice were more effective at protecting from diabetes compared with DCs from vehicle-treated mice (Figure 6E) (log-rank (Mantel–Cox) test, *p* < 0.0001).

### 3.8. A Salmonella-Based Vaccine Increases the Expression of the Autoimmune Inhibitory Genes

**Figure 7:**
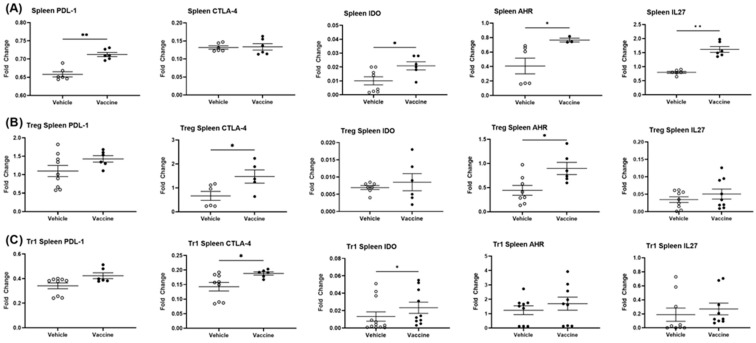
A *Salmonella*-based vaccine increases expression of immune inhibitory genes. Thirty days post-treatment, the fold changes in mRNA gene expressions of CD274 (PDL-1), CTLA-4, IDO, AhR, and IL27 in pooled splenocytes (**A**), Treg-sorted splenocytes (**B**), and Tr1-sorted splenocytes (**C**) isolated from vaccine- and vehicle-treated mice were determined. The data displayed are the average of the fold changes ± SD from two independent experiments. Statistical analysis using Mann–Whitney *t* test shows significance between vaccine- and vehicle-treated mice (* *p* < 0.05, ** *p* < 0.01).

**Figure 8:**
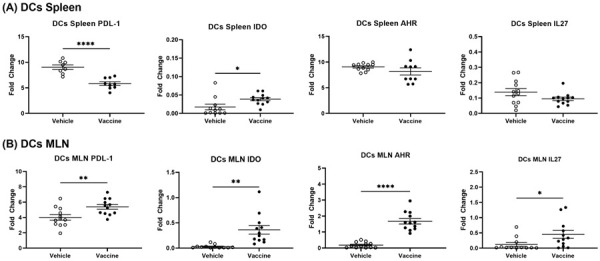
mRNA levels of immune regulation molecules were increased more in MLN versus splenic DCs from vaccine-treated NOD mice. Vaccine or vehicle were given to NOD mice and a month later DCs were obtained. Relative amounts of CD274 (PDL-1), CTLA-4, IDO, AhR, and IL27 mRNA from splenic (**A**) and MLN DCs (**B**). Data are the means ± SD from 2 independent experiments. The Welch’s *t* test showed significance (* *p* < 0.05, ** *p* < 0.01, **** *p* < 0.001).

Immune inhibitory surface proteins such as CTLA4, CD47, and SIRPα, among many others, provide a means for limiting autoimmunity. These cell surface receptors can be found on many immune cell types including T, NK, and dendritic cells and macrophages [53] but are in certain cases also found in non-immune cells. Expression levels of mRNAs in CD11c^+^ (DCs), CD4^+^ CD25^+^ Tregs, and CD4^+^ CD49b^+^ Tr1 cells from spleens of vaccine- and vehicle-treated mice were determined. PDL-1, IDO, AhR, and IL27 mRNA were increased in splenocytes from vaccine- compared to vehicle-treated animals (Figure 7A). Splenic Tregs and Tr1 cells from vaccine-treated mice displayed significantly more CTLA-4, AhR, PDL-1, and IDO mRNA compared to cells from vehicle-treated animals (Figure 7B,C). Splenic DCs from vaccine-treated mice showed less PDL-1, upregulated IDO, and no changes in AhR or IL27 mRNA compared to cells from vehicle-treated mice (Figure 8A). MLN DCs from vaccine-treated mice showed increased levels of PDL-1, IDO, AhR, and IL-27 mRNA in comparison to cells from vehicle-treated mice (Figure 8B). Splenic DCs from vaccine-treated mice showed increased DC-SIGN mRNA (dendritic cell-specific intercellular adhesion molecule-3–grabbing non-integrin; CD209A) compared to cells from vehicle-treated animals (Figure S6). DC-SIGN is associated with the inhibition of inflammation and is found primarily on DCs and some macrophages [54].

## 4. Discussion

Oral antigen-based vaccines induce tolerance via an adaptive immune response [55]. Plasmid DNA vaccines are, for the most part, safe but lack the control and persistence of antigen expression [56,57,58]. In mice, plasmid DNA encoding beta-cell autoantigens prevented the progression of T1D [59,60]. A *Salmonella*-based vaccine provided efficient delivery of autoantigens and could accommodate combinations of autoantigens and immune modulators for more specific therapy that is safe and perhaps cheaper [9,10,11,12]. Other means of limiting or correcting T1D include giving rapamycin [61], pluripotent stem cells [61,62], T-cell exhaustion [63], antigen-specific therapies [64,65], dipeptidyl peptidase-4 inhibitors, and proton-pump inhibitors [66]. An engineered plasmid of *Salmonella* showed promise in treating T1D in NOD mice through immune modulation mechanisms [9,10,11,12,13,14]. The approach herein used live attenuated engineered *Salmonella* for the delivery of two immunosuppressive cytokines to promote tolerance in tissue resident T-cells and APCs.

Cytokines can control the recruitment of immune cells. As an example, oral *Lactococcus* secreted IL10 to limit islet infiltration by immune cells and prevent and halt the progression of diabetes [67,68,69]. Similarly, our oral *Salmonella* vaccine induced tolerance, in part, by elevating IL10, IL2, IL13, and MCP-1 and lowering IFNγ, GM-CSF, TNFα, IL7, and IL12, in responder animals. The increase in the IL10 levels is probably not the result of IL10 being over-expressed by the *Salmonella* vector as the IL10 levels were measured in the serum of mice 3 months after vaccine treatment. By this time, *Salmonella* was cleared from the animals, indicating that the increase in IL10 level was from immune cell regulation and the maintenance of immunological tolerance [11,13]. IL10 has anti-inflammatory activity and induces long term Tr1 cells that then provide lasting tolerance [61,70,71]. Of note, IL10, TGFβ, and MCP-1 induced tolerogenic properties in effector/memory T-cells and macrophages in individuals with T1D [33,72,73]. IL2 is considered a tolerogenic cytokine as it prevents autoimmune diseases by promoting the differentiation of certain immature T-cells into regulatory T-cells [74,75] and also promotes beta-cell proliferation [33]. Indeed, autoimmune diabetes is associated with defects in the IL2 signaling pathway [76].

Correspondingly, antigen-specific suppressor Tregs, Tr1 cells, and tol-DCs, all of which inhibited responder T-cell proliferation, were observed in lymphoid organs from vaccinated mice. Changes in inhibitory genes in Tregs and tol-DCs may partly explain the immunosuppressive efficacy of the cells. As an antigenic determinant, proinsulin was incorporated in the *Salmonella* plasmid and was recognized by effector T-cells. When expressed in APCs, it induced immune tolerance [77]. Cross talk between DCs and Tregs in autoimmune and chronic inflammatory diseases remains a point of debate. Tregs, as a sink for IL2 secretion, may trigger immune tolerance via the secretion of anti-inflammatory cytokines or via cell-to-cell contact [78]. Tolerogenic semi-mature DCs induced Tregs [79,80,81,82]. Additionally, adoptive therapy using Tregs alone suppressed immune cells [83]. The debate about which cell type triggers the other continues. However, interplay between cell types suggests a bidirectional loop for the inhibition of chronic inflammatory disease [84].

Controlling T1D may be possible through augmenting endogenous regulatory mechanisms [85]. Autoimmune diabetes originates from the imbalance between T-effector cells and Treg cells, with a failure to maintain self-tolerance [86,87]. In this study, the highest level of regulatory cells was observed in the lymphatic organs of animals treated with the vaccine, providing a correlation between regulatory cell induction and prevention and the reversal of diabetes. The induction of Tr1 cells occurred at the same time as Tregs and was linked to the *Salmonella* vaccine. In autoimmune diseases, the interaction between anti-inflammatory cytokines and Tregs regulates disease progression [88]. Tregs inhibited effector T-cell proliferation in a ratio-dependent manner [89]. IL10 from Tr1 cells and Foxp3^+^ Tregs was altered in NOD mice [90]. Tr1 cells, likely arising from memory and total CD4 T-cells, controlled effector T-cells via IL10 signaling to limit the development of diabetes [91]. Further, adoptive transfer of CAR Tregs prevented diabetes in NOD mice [92].

In vitro and in vivo data suggest that DCs are important in immune suppression [93,94,95,96,97,98]. Because DCs are found in different lymphoid organs, they can initiate different adaptive pathways. These are dependent on DC phenotype [99]. DCs are classified into three major subsets. Conventional DCs (cDCs), also known as myeloid DCs (CD11c^+^ CD11b^+^), cross-present extracellular antigens and stimulate CD8 T-cells [100,101,102]. Plasmacytoid DCs (pDCs) (CD11c^interm^ B220^+^) link innate and adaptive immunity and secrete pro-inflammatory interferons in response to viruses [102,103,104,105]. Lymphoid DCs (CD11c^+^ CD8^+^) migrate to various locations including secondary lymph nodes to process antigens and induce tolerance [102,106]. DCs in the gut PPs promote the differentiation of T helper-2 cells and Tregs as well as secrete TGFβ [107,108,109]. DCs were found to be a maintenance factor for Tregs [52]. Indeed, when naïve T-cells were stimulated with antigen pulsed-DCs in the presence of TGFβ, the DCs assumed a tolerogenic phenotypic [110]. IL10-conditioned DCs stopped the onset of diabetes in NOD mice [87]. In addition, tol-DCs eliminated autoreactive CD8^+^ T-cells, caused clonal deletion and anergy, and converted peripheral T-cells subsets into regulatory T-cells [111]. In the present study, tol-DCs exhibited an immature DC phenotype, low MHC class II, induction of Tregs, expression of PD-L1 and PD1, alterations in chemokine receptors, decreased pro-inflammatory cytokine secretion, and enhanced immunoregulatory cytokine secretion [111,112,113,114].

In this study, a *Salmonella*-based vaccine increased expression of the immune checkpoint molecules CTLA-4 and PDL-1. Additionally, there was increased expression of the aryl hydrocarbon receptor (AhR) in vaccine-treated mice. The AhR is a transcriptional factor that suppresses the expression of pro-inflammatory cytokines and attenuates autoimmune responses in T1D [115]. Furthermore, the *Salmonella*-based vaccine increases the expression of indoleamine 2,3-dioxygenase (IDO) in DCs. This is important as the secretion of IDO induces tolerance [116,117,118]. While the increased expressions of CTLA-4, PD-L1, AhR, and IDO may contribute to the observed reduction in diabetes-related inflammation, it is worth noting that the overexpression of these molecules can potentially suppress the immunogenic function of T-cells and, in some cases, induce T-cell apoptosis [44]. Interestingly, the vaccine increased DC-SIGN and this modulates DC function, interacts with immune checkpoint molecules, and facilitates the presentation of antigens to promote immune tolerance [105], especially in situations where self-antigens need to be tolerated [119]. Furthermore, the *Salmonella*-based vaccine increased IL27, which is produced by DCs and macrophages in response to infection or inflammation [120]. IL27 may play a role in modulating the immune response to prevent or limit autoimmune attack on beta-cells [121]. Additionally, IL27 enhanced the development and function of Tregs in T1D [122]. This suggests that the vaccine created an immunosuppressive microenvironment that limited the autoimmune response without compromising overall immune function.

## 5. Conclusions

The administration of a *Salmonella* vaccine to mice predisposed to T1D restored glucose balance. This occurred in association with increased Tregs, Tr1 cells, and DCs. Additionally, the regulatory cells from the vaccine-treated mice were antigen-specific and showed increased profiles of cytokines useful in controlling inflammation. Decreases in levels of pro-inflammatory cytokines IFNγ, GM-CSF, TNFα, IL6, and IL12 likely also helped. Other changes in the range of anti-inflammatory molecules were noted. Additional studies may determine the necessary and sufficient changes that underlie vaccine-merited improvements in T1D.

## Data Availability

Data are contained within the article.

## Supplementary Materials

The following supporting information can be downloaded at: https://www.mdpi.com/article/10.3390/vaccines12030276/s1, Figure S1: Effect of A *Salmonella*-based vaccine on serum cytokines; Figure S2: A *Salmonella*-based vaccine does not alter immune response against *Salmonella*; Figure S3: Gating strategy for regulatory T-cells; Figure S4: A *Salmonella*-based vaccine increases functional Tregs; Figure S5: Gating strategy for subsets of DCs; Figure S6: A *Salmonella*-based vaccine increased expression of DC-SIGN (CD209A).
