# Supplementary material for: Mechanism of Action of Oral Salmonella-Based Vaccine to Prevent and Reverse Type 1 Diabetes in NOD Mice
Source: Vaccines (Basel). 2024 Mar 6;12(3):276. doi: 10.3390/vaccines12030276 (PMC10975319; doi:10.3390/vaccines12030276)
Supplement: Supplementary file 1 [file vaccines-12-00276-s001.zip › vaccines-2884497-supplementary.pdf]

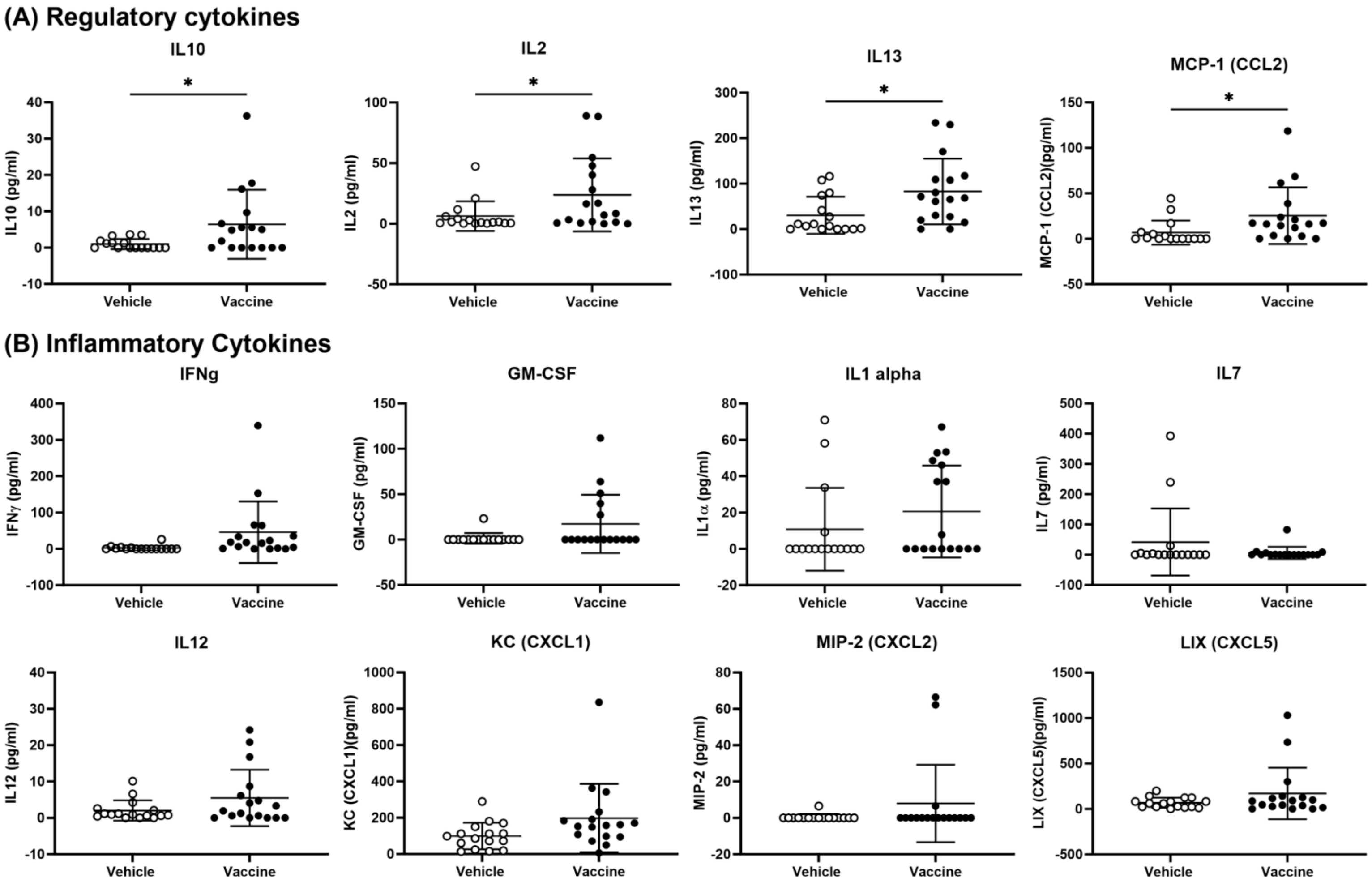

Figure S1. Effect of A *Salmonella*‒based vaccine on serum cytokines. Serum was collected from vaccine‒ and vehicle‒treated mice and cytokines were quantified using a Multiplex Assay. (A) Levels of regulatory cytokines IL10, IL2, IL13, and CCL2. (B) Levels of pro‒inflammatory cytokines IFNγ, GM-CSF, IL1α, IL7, IL12, CXCL1, CXCL2, and CXCL5. Data presented as means ± SD from vaccine (*n*=17) and vehicle-treated mice (*n*=16). Significant differences between vaccine‒ and vehicle‒treated were determined by the unpaired t test (*$p < 0.05$).

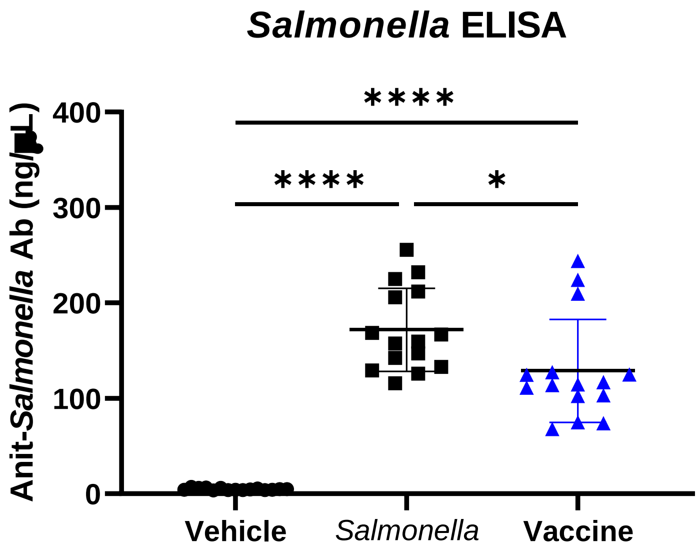

Figure S2. A *Salmonella*–based vaccine does not alter immune response against *Salmonella*. Serum was collected from vaccine- and vehicle-treated and *Salmonella*–infected mice and anti–*Salmonella* antibody was quantified using ELISA. Data presented as the means ± SD from 2 independent experiments. Significant differences between vaccine– and vehicle–treated and *Salmonella*-infected were determined by one-way ANOVA (* $p<0.05$; **** $p<0.001$).

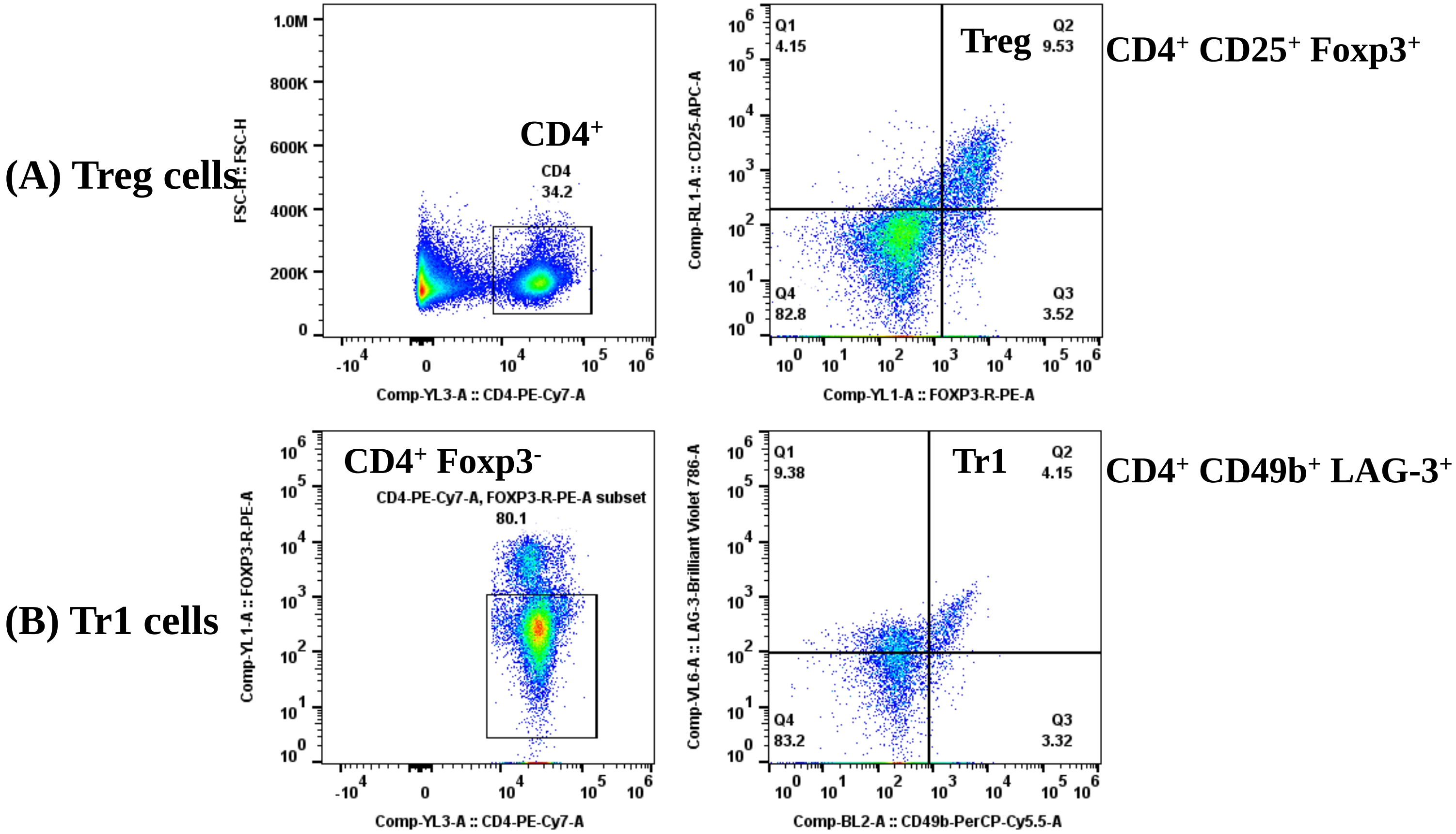

Figure S3. Gating strategy for regulatory T–cells. Single cell suspensions of lymphoid organs were prepared on day 30 post-vaccination. **(A)** Representative FACS plots gated on live $CD4^+$ T–cells indicate $CD4^+$ $CD25^+$ $Foxp3^+$ (Treg) cells. **(B)** Representative FACS plots gated on live $CD4^+$ $Foxp3^-$ T–cells indicate $CD4^+$ $CD49b^+$ LAG-3^+ (Tr1) cells.

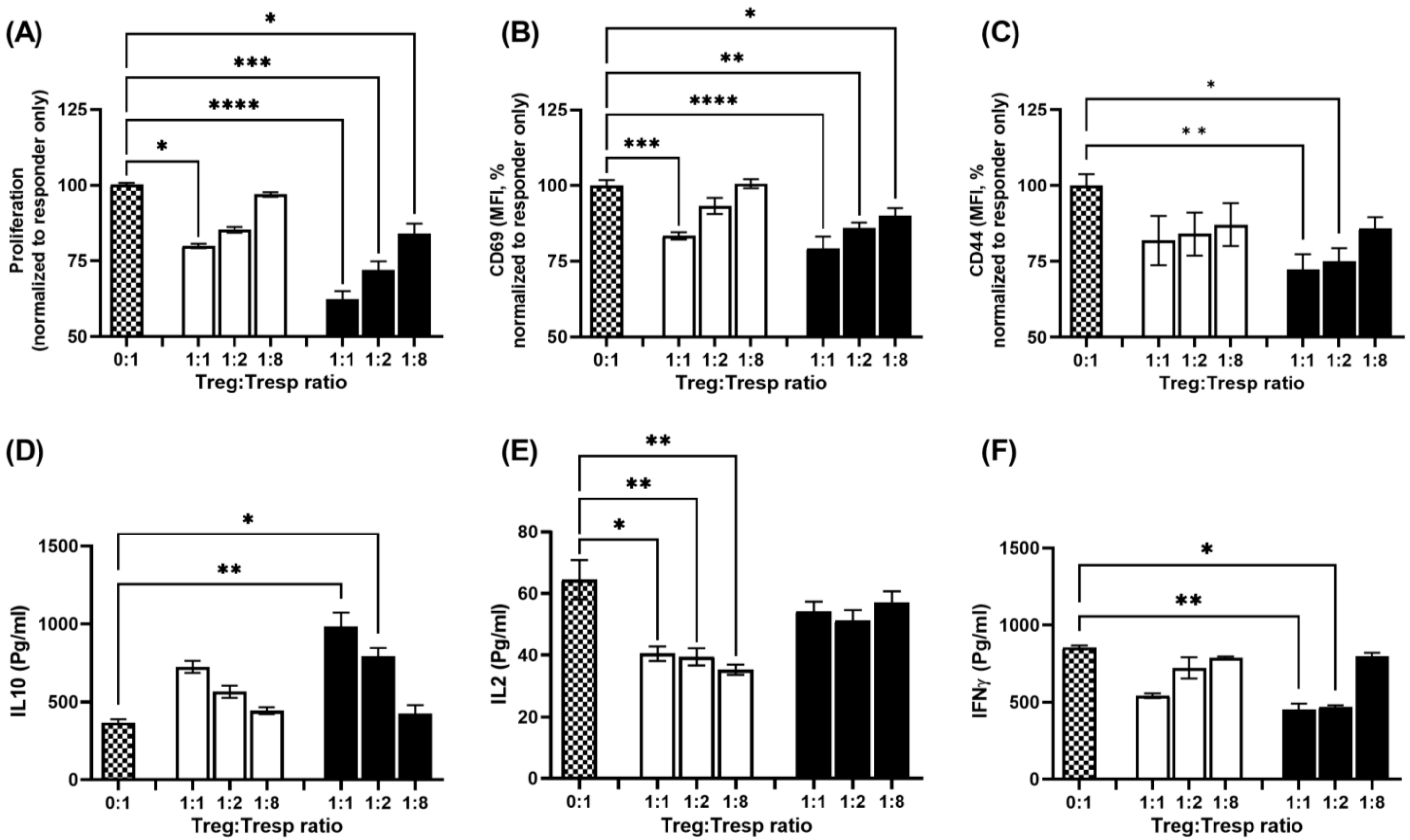

Figure S4. A *Salmonella*−based vaccine increases functional Tregs. $CD4^+CD25^-$ responder T cells (Tresps) were isolated from normoglycemic NOD mice, dye labeled, and pulsed with 10 µg/ml insulin $B_{9\text{-}23}$ peptide. Splenic APCs were co−cultured at different ratios of $CD4^+CD25^+$ Tregs for 4 days. **(A)** Proliferation of Tresps, shown as percentage of cells that had undergone one or more divisions, normalized to proliferation by Tresps alone. Activation of Tresps shown as a percentage of CD69 **(B)** or CD44 **(C)** expression, normalized to the percentage of Tresps alone. Measurement of cytokines, IL10 **(D)**, IL2 **(E)**, and IFNγ **(F)** in the culture supernatants by ELISA assay. Data presented as the means ± SD from 2 independent experiments. Significant differences between vaccine- and vehicle-treated mice were determined by one−way ANOVA (* $p<0.05$, ** $p<0.01$, *** $p<0.005$, **** $p<0.001$). Hashed, white, and black bars represented Tresp alone, Treg from vehicle, and Treg from vaccine, respectively.

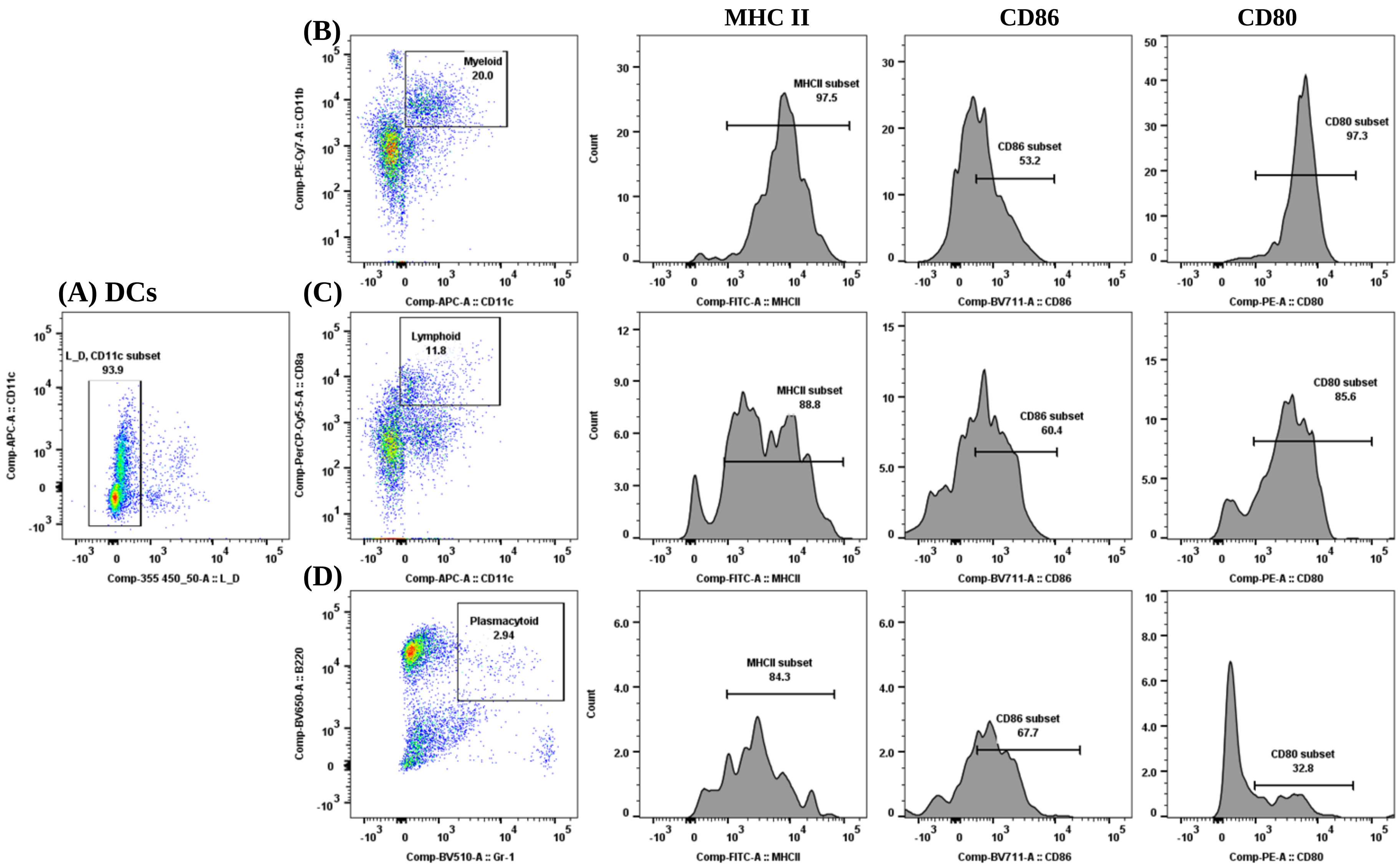

Figure S5. Gating strategy for subsets of DCs. Single cell suspensions of lymphoid organs were prepared on day 30 post–vaccination. **(A)** Representative FACS plots gated on live CD11c indicate CD11c^{+} cells. **(B)** Representative FACS plots gated on live CD11c indicate CD11c^{+} CD11b^{+} for myeloid DCs and gated for MHCII, CD86, and CD80. **(C)** Representative FACS plots gated on live CD11c indicate CD11c^{+} CD8^{+} for lymphoid DCs and gated for MHCII, CD86, and CD80. **(D)** Representative FACS plots gated on live CD11c indicate CD11c^{+} B220^{+} Gr1^{+} for plasmacytoid DCs and gated for MHCII, CD86, and CD80.

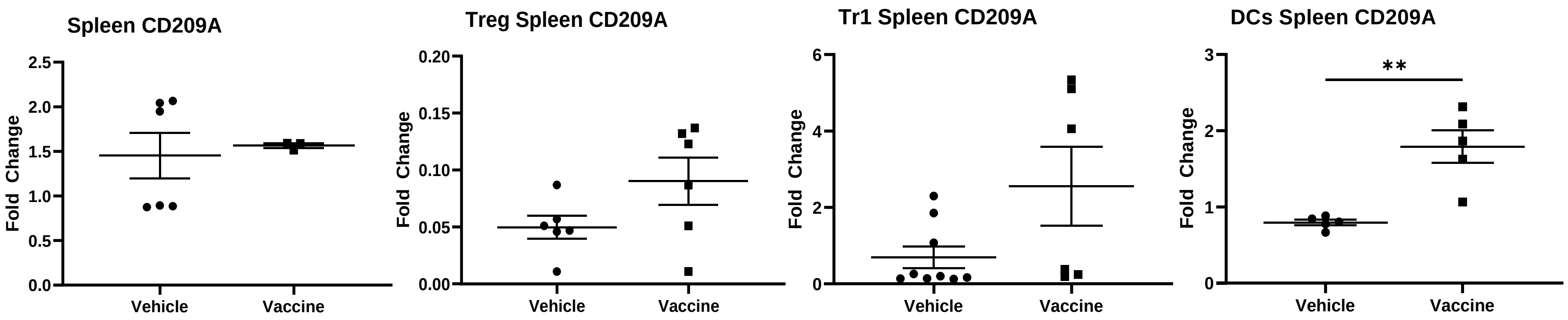

Figure S6. A *Salmonella*‒based vaccine increased expression of DC-SIGN (CD209A). Thirty days post‒vaccination the fold changes in gene expression of DC-SIGN (CD209A) in splenocytes, Treg, Tr1, and DCs from splenocytes isolated from vaccine- and vehicle-treated mice were determined. Data presented as the means ± SD from 2 independent experiments. Statistical analysis using Welch's t test shows significance between vaccine‒ and vehicle‒treated mice (** $p < 0.01$).
